# Effect of Different Administered Doses of Capsaicin and Titanium Implant Osseointegration

**DOI:** 10.3390/medicina60071094

**Published:** 2024-07-04

**Authors:** Muhammet Bahattin Bingül, Mehmet Gul, Serkan Dündar, Kevser Sökmen, Gökhan Artas, Mehmet Emrah Polat, Murat Tanrisever, Erhan Cahit Ozcan

**Affiliations:** 1 Department of Oral and Maxillofacial Surgery, Faculty of Dentistry, Harran University, Sanliurfa 63300, Turkey; bahattinbingul@gmail.com (M.B.B.); mehmetemrpolat@gmail.com (M.E.P.); 2 Department of Periodontology, Faculty of Dentistry, Harran University, Sanliurfa 63300, Turkey; 3 Department of Periodontology, Faculty of Dentistry, Firat University, Elazig 23119, Turkey; dtserkandundar@gmail.com; 4 Department of Periodontology, Faculty of Dentistry, Alanya Alaaddin Keykubat University, Antalya 07070, Turkey; kvsrkrz132@gmail.com; 5 Department of Medical, Faculty of Medicine, Pathology Firat University, Elazig 23119, Turkey; gartas79@yahoo.com; 6 Department of Surgery, Faculty of Veterinary Medicine, Firat University, Elazig 23119, Turkey; mtanrisever@firat.edu.tr; 7 Department of Esthetic, Faculty of Medicine, Plastic and Reconstructive Surgery, Elazig 44090, Turkey; erhancahitozcan@gmail.com

**Keywords:** capsaicin, oxidative stress, osseointegration, bone implant connection

## Abstract

*Background and Objectives*: This study aimed to evaluate the histological and biochemical effects of capsaicin on implant osseointegration and oxidative stress. *Materials and Methods*: Male Wistar albino rats weighing between 250 and 300 g were used in this study. Twenty-four rats were randomly divided into three equal groups: implant + control (*n* = 8), implant + capsaicin-1 (*n* = 8), and implant + capsaicin-2 (*n* = 8). Additionally, 2.5 mm diameter and 4 mm length titanium implants were surgically integrated into the corticocancellous bone parts of the femurs. In the treatment groups, rats were injected intraperitoneally with 25 mg/kg (implant + capsaicin-1) and 50 mg/kg (implant + capsaicin-2) of capsaicin. No additional applications were made in the control group. Three rats in total died during and after the experiment as a result of the analyses performed on 21 animals. *Results*: The highest total antioxidant status value was found in capsaicin dose 2, according to the analysis. The control group had the highest total oxidant status and oxidative stress index values, while group 2 of capsaicin had the lowest. After analysis, we found that there was no observed positive effect on osteointegration in this study (*p* > 0.05), although the bone implant connection was higher in the groups treated with capsaicin. *Conclusions*: A positive effect on osteointegration was not observed in this study. This may be due to osteoclast activation. However, it was found that it has a positive effect on oxidative stress. Osteoclast activation may be the cause of this phenomenon. Capsaicin was found to have a positive effect on oxidative stress (*p* < 0.05). It was also observed to have a positive effect on oxidative stress.

## 1. Introduction

According to this report, sympathetic and sensory nerves play a significant role in bone metabolism. Studies have shown that sympathetic nerves have an impact on both bone formation and resorption [1,2,3,4,5]. In this interaction, the sensory neuropeptide calcitonin gene-related peptide (CGRP) molecules regulate osteoclastogenesis [4,5,6]. Studies have reported that neural tissues play a role in regulating blood flow and bone metabolism. Therefore, they are involved in bone remodeling metabolism [7,8]. Regions with higher bone metabolism have more nerve endings, which can aid in bone reconstruction by influencing bone metabolism [9,10].

Studies have reported that capsaicin is a neurotoxic agent and acts on sensory neurons through transient receptor potential vanilloid 1 (TRPV1) molecules [11,12]. As a result, TRPV1 is activated, and neurotoxicity has been reported as a result of the accumulation of calcium and sodium cations [13]. Low-dose capsaicin administration has been reported to play a role in pain sensation due to TRPV1 activation. According to this report, the application of high doses of capsaicin has a neurotoxin effect, affecting massive ion flow [13,14]. Studies have reported that dorsal root neurons have losses in the immunoreactivity of CGRP when high capsaicin is administered [14,15,16]. A long-term blocking effect on sensory receptors has been reported from systemic administration of capsaicin [17].

In the studies, nerve endings on the implant surface were evaluated, and as a result, bone neurofilament protein (NFP) was detected in an area of 200 mm. However, the exact role of these structures has not been determined [18,19]. It has not been fully determined whether these structures have any function in implant osseointegration or whether they play a role as a regulator in bone metabolism [20].

In one study, capsaicin was administered to newborn rats. When the rats became adults, tooth extraction was performed, and a 21% reduction in alveolar bone resorption was observed in the capsaicin-treated group compared to the non-capsaicin-treated group [21]. In a rat study, capsaicin was administered, and as a result, a 40% decrease in bone resorption was detected in adult rats. In the analysis, a decrease in the resorption surface and the number of osteoclasts were determined [15]. Contrary to these studies, opposite situations were found in some studies. For example, Offley et al. In the study conducted by et al., it was reported that it decreased trabecular integrity and increased osteoclast count in bone metabolism [16].

Studies have reported that implant success is determined by the immobility of the implant and the quality of osseointegration. Branemark et al. coined the term ‘implant osseointegration,’ which refers to a direct functional and structural connection between the implant and bone [22]. Implants can prevent functional loss resulting from tooth loss. Implants are composed of a variety of materials, each with unique designs and surface properties [23].

Capsaicin is assumed to have antioxidant properties. It can prevent lung emphysema and the development of free radical-related injuries, such as autoimmune nephrosis and muscular dystrophy [24]. Pulmonary emphysema and asthma share a wide range of symptoms and clinical signs that can hinder differential diagnosis. TRPV1 plays an important role in coughing and airway inflammation. As a consequence, the TRPV1 agonist capsaicin is of great interest in investigating the cellular effects and regulatory pathways mediating these respiratory conditions [25].

Oxidative stress is recognized as a contributing factor to the development of these diseases. Reactive oxidants can trigger free radical reactions that can have negative effects on the human body. A study on rats suggested that capsaicin may have a therapeutic effect on carbohydrate and lipid metabolism as well as oxidative stress. However, more research is needed to confirm these findings [24].

Titanium implants are a commonly used medical device in craniomaxillofacial surgery, as well as in human and veterinary orthopedics. In the literature, the effects of many biological substances on the application of titanium implants have been investigated. This study aimed to examine the effects of systemic administration of capsaicin at different doses on oxidative stress and bone–implant connection parameters after surgical integration of the implants in rat tibias.

## 2. Materials and Methods

### 2.1. Animals and Study Design

This study was approved by Harran University’s (2019-006-03, approval date: 7 November 2019) Local Ethics Committee on Animal Experiments. It was carried out at the Experimental Research Center of Harran University, and the Helsinki Declaration rules were strictly followed during the experiments. The Wistar albino rats used in the experiments were obtained from the Experimental Research Center of Harran University. In these studies, experiments were carried out without causing suffering to animals, and all ethical rules were respected.

The number of animals was determined by a power analysis with an alpha error of 0.05 and a beta error of 0.20, and it was determined that a minimum of 21 rats were required for the study. This study was started with a total of 24 rats, 8 rats in each group, in order to ensure accurate statistical analysis in case the rats died during the experimental stages. This study aimed to evaluate and histopathologically examine the effect of capsaicin on the osteointegration of dental implants after titanium implants were integrated into the tibia of rats.

Implant + control group: The joint capsule was opened in the location where the right femur and tibia meet, and titanium-manufactured machined surfaced implants with a 2.5 mm diameter and 4 mm length were integrated. The rats were systemically given saline (daily intraperitoneally during the experimental period).

The same surgical procedure was applied to the rats in the implant + capsaicin dosage 1 and implant + capsaicin dosage 2 groups. Systemically, capsaicin was administered 25 mg/kg and 50 mg/kg intraperitoneally to rats daily during the experiment, respectively.

Eight rats from each group were euthanized and sacrificed on the 28th day with the taking of blood. After the experimental procedures, the samples, which were implants surrounded by bone tissues, were fixed in a buffered formaldehyde solution for 72 h.

### 2.2. Surgical Procedures

Before the surgical procedures, Ketamine Hydrochloride (Ketamidor-Richter Pharma, Austria) 10 mg/kg and Xylazine (Rompun-Bayer, Germany) 50 mg/kg injections were administered intramuscularly to all subjects for anesthesia, following the rules of asepsis and antisepsis. After the operation area was cleaned with Povidone Iodine (Batticon-Adeka, Turkey), it was covered with sterile drapes, leaving the operation area exposed. For local hemostasis, 0.5 cc, 4% articaine (Ultracain DS-Aventis, Istanbul, Turkey) containing 0.006 mg/mL epinephrine was applied to the operation area. A full-thickness flap was opened with a no. 15 scalpel. The joint capsules of the rats were exposed with a median made to the junction of the incision of the tibia and femur. After the corticocancellous bone, part of the femur bone, where it articulates with the tibia, was reached.

Implant beds of 2.5 mm in diameter and 4 mm in length were created in the corticocancellous bone portion of the right femur of each rat where it meets the tibia and opened with a drill with saline irrigation. Titanium implants, 2.5 mm in diameter and 4 mm in length, with machined surfaces, were integrated into the cavities at the bone level. All subjects received 50 mg/kg cefazolin sodium intramuscularly as an antibiotic for 3 days postoperatively to prevent infection. For pain relief, 1 mg/kg of tramadol hydrochloride was given intramuscularly for 3 days postoperatively. All wound sites were sutured, primarily. The operation site was sutured using a 3.0 silk suture. Postoperative specimens were preserved in formaldehyde and sent to pathology for histologic analysis.

### 2.3. Histopathological Analysis

After the experiments, blood serum samples were collected from the heart for biochemical oxidative stress analysis. In addition, bone tissues were cut with the implant, and histopathological samples were obtained for bone–implant connection. Samples were fixed in 10% neutral formaldehyde before histological analysis. Before hard tissue analysis, decalcification was performed using an EDTA solution. In this way, the samples were made soft. The samples, which were passed through the alcohol series required for the analysis, were dehydrated, cleaned, and embedded in paraffin. Sagittal sections were obtained using a microtome blade (5–6 µm) for the areas to be analyzed. Afterward, hematoxylon–eosin staining was performed and examined using a light microscope. The stained histological preparations were left to dry overnight, after which the sample surfaces were covered with a coverslip of methyl methacrylate. Digital images of all samples were taken at 4× magnification with a digital camera (Olympus DP 70, Tokyo, Japan) connected to a light microscope (Olympus BX50). Histological bone healing was assessed using the Image J Analysis Program (Image J version 1.44; National Institutes of Health, Bethesda, MD, USA).

### 2.4. Biochemical Analysis

In biochemical analysis, the kit protocol of the manufacturer was applied. Then, a microplate reader was used for value reading (Cytation-1, Biotek, USA). The oxidative stress index was obtained by the ratio of the total oxidant status value with the total antioxidant status value [26,27]. Trolox was used as the calibrator material in the analysis. Trolox is known as a water-soluble vitamin E analog. Blood samples taken for oxidative stress analyses were kept at −80 degrees. In the analysis, oxidative stress parameters, total oxidative status (TOS), total antioxidant status (TAS), and oxidative stress index (OSI) were evaluated.

### 2.5. Semi-Quantitative Scoring of Histopathologic Parameters

A semi-quantitative score was determined by examining the cells in the bone tissue, such as osteoblast, osteocyte, and osteoclast. In the evaluation of histological sections, fifteen different areas were scanned for each slide, and then the average value of ten randomly selected cells was determined. From these averages, ten points were obtained for each animal group, and then these values were analyzed statistically. The method we used has also been used in previous histochemical studies of bone tissue [28,29,30].

### 2.6. Statistical Analysis

While evaluating the data in the research, a statistical analysis program from IBM SPSS Statistics 22 was used. Kolmogorov–Smirnov and Shapiro–Wilks tests were used to test whether the data in the study were normally distributed. In the analysis of data that were found to be normally distributed, a one-way ANOVA was used to identify the group that caused the difference, and Tukey’s HSD test was used for comparisons between groups. In the analysis of data that were not normally distributed, the Kruskall–Wallis test was used to identify the group that caused the difference, and the Mann–Whitney U test was used for comparisons between groups. Statistical significance was evaluated at the *p* < 0.05 level.

## 3. Results

Three rats (1 animal from each group) in total died during and after the experiment as a result of the analyses performed on 21 animals. In Table 1, the TAS value was 1.19 ± 0.15 in the only implant-integrated group, 1.38 ± 0.09 in the capsaicin dosage 1 group, and 1.45 ± 0.08 in the capsaicin dosage 2 group. These differences were also statistically significant (*p* < 0.05). In Table 1, the TOS value was 15.21 ± 1.35 in the control animals, 11.81 ± 0.65 in the capsaicin dosage 1 group, and 11.11 ± 0.88 in the capsaicin dosage 2 group. These differences were also statistically significant (*p* < 0.05). In Table 1, the OSI value was 1.3 ± 0.2 in the controls, 0.87 ± 0.05 in the capsaicin dosage 1 group, and 0.77 ± 0.07 in the capsaicin dosage 2 group. After the analysis, significant differences were obtained between the control group and the capsaicin groups (*p* < 0.05). However, no significant differences were found between the capsaicin treatment groups (*p* > 0.05).

**Figure 1:**
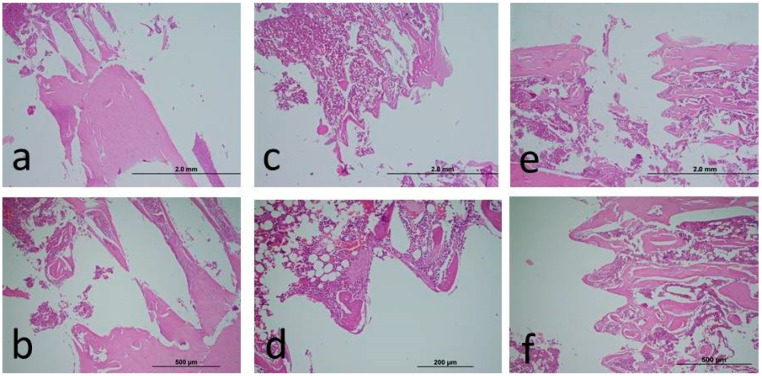
Decalcified histologic images of peri-implant bone tissues of (**a**): Control, (**b**): Capsaicin Dosage 1 (25 mg/kg), (**c**): Capsaicin Dosage 2 (50 mg/kg) (4× magnification, Hematoxilen Eosine, X: 10 times). Decalcified histologic images of peri-implant bone tissues of (**d**): Control, (**e**): Capsaicin Dosage 1 (25 mg/kg), (**f**): Capsaicin Dosage 2 (50 mg/kg) (10× magnification, Hematoxilen Eosine, X: 10 times).

As a result of the analysis, although the BIC was higher numerically in the capsaicin-applied groups, no significant difference could be obtained (*p* > 0.05). In Table 2, the ratio of osseointegration between the capsaicin treatment groups was measured. In the statistical analysis between the groups, although numerical osseointegration values were higher in experimental groups compared to controls, no statistical difference was found in terms of capsaicin-administered groups’ percentage values when compared with the controls (*p* > 0.05) (Figure 1).

**Figure 2:**
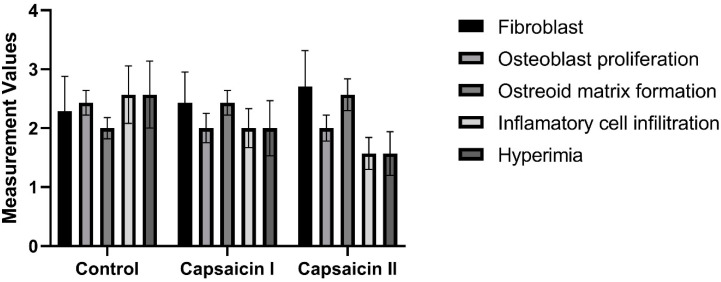
Distributions of measurement values by groups.

There is no statistically significant difference between groups in terms of fibroblast, osteoblast proliferation, osteoid matrix formation, inflammatory cell infiltration, and hyperemia measurement values (*p* > 0.05) (Table 3; Figure 2).

In Table 2, the percentage values of capsaicin administered to the rats were measured. In the statistical analysis between the groups, no statistically significant difference was found in terms of capsaicin percentage values (*p* = 0.134).

## 4. Discussion

Lucke et al. investigated the osteointegration of titanium implants in a study. The researchers conducted and evaluated the study on days 7, 14, 21, 28, 35, and 42 [31]. In this study, the osseointegration levels of titanium implants were evaluated at day 28 to assess early osteointegration, which is important in implant-supported prosthetic treatment. Offley et al. found that sensory neurons function in bone remodeling and that this event is regulated by transmitters released from peripheral nerve terminals [16].

In treatments using capsaicin, osteoclast number increases while bone volume, osteoblast number, and activity decrease [16]. In another study, 37.5 mg/kg capsaicin was shown to not affect bone remodeling, while 150 mg/kg capsaicin was reported to increase TRAP 5b levels in plasma. However, since the osteocalcin concentration in the plasma is not affected, it has been claimed to contribute positively to sensory nerve innervation [16,17]. In our study, capsaicin was applied at different doses (25 mg/kg and 50 mg/kg), and positive effects on bone implant fusion were observed. However, no significant difference was obtained. There are in vivo studies showing the effect of the sensory nervous system on bone tissue metabolism [5,17]. In a study where 150 mg/kg of capsaicin was applied, it was reported that the strength increased in the proximal tibia but the amount of trabecular bone decreased. In addition, high-dose capsaicin (150 mg/kg) has been claimed to increase bone fragility due to its support for osteoclast development [17]. Osseointegration means the adhesion of new bone to the implant surface without any fiber tissue [32]. Therefore, bone implant connection and bone metabolism in peri-implant tissues are closely related. Three weeks after implantation, the areas of bone around the implant in the sensory denervated group were lower than in the sensory non-denervated group. It has also been reported that sensory denervations can reduce the activity of osteoblasts that support bone formation [16,17]. A study reported that capsaicin induced small dorsal root ganglion cells, leading to the loss of unmyelinated sensory axons. Other studies have shown that CGRP inhibits osteoclastogenesis and bone resorption while also stimulating osteoblast proliferation and bone formation [33,34,35].

Capsaicin has been reported to cause an increase in CGRP release from cardiac C-fiber nerve endings in the heart [36]. In particular, the use of calcitonin receptor-like receptor/receptor activity-modifying protein 1 receptors (CLR/RAMP1) not only caused vascular relaxation resulting from CGRP signaling but also modulated inflammation by regulating proinflammatory cytokine production in dendritic cells [37]. Histologic evaluations revealed a significant reduction in inflammation in the capsaicin-treated groups. We suggest that the effect of capsaicin on CGRP release regulates proinflammatory cytokine production in dendritic cells.

Zhang et al. reported that capsaicin-sensitive sensory nerve fibers were CGRP-positive nerve fibers [17]. In addition, it has been reported that when regular capsaicin treatment is applied, CGRP-positive nerve fibers are depleted, bone formation is inhibited, and bone resorption is increased [38].

In the study by Zhang et al., a significant decrease in density in tibia cancellous bone mass was observed after capsaicin treatment. This shows that normal mechanical loading does not lead to a normal balance of bone remodeling when capsaicin is administered. It has also been suggested that hindlimb suspension (HLS) does not cause additional bone loss after capsaicin treatment depletes CGRP-positive nerve fibers [17].

In light of this information, in this study, 25 and 50 mg/kg of capsaicin were administered systemically, and no significant difference could be obtained between the groups. Capsaicin has a similar effect on bone metabolism in implant osseointegration. Apart from that, capsaicin appears to be a potent antioxidant for preventing in vivo free radical reactions. It was concluded in vivo that capsaicin consumed for several days might have a beneficial and potential therapeutic effect [24]. In this study, the oxidative stress values of the capsicin-applied groups were lower than the control group. A significant difference between the groups was reported. This shows us that systemic capsaicin has antioxidant properties. Sheppard et al. reported in 2022 that free oxygen radicals play an important role in the fracture healing process and showed that alpha-tocopherol, which has antioxidant properties, contributes to fracture healing by suppressing free oxygen radicals [39].

The study reported that capsaicin is a powerful antioxidant through different mechanisms [40]. In the study conducted by Chaudhary et al., statistically significant differences were obtained in the antioxidant levels in the plasma of rats administered capsaicin. As a result, it is reported that capsaicin functions to protect cells from oxidative stress damage [41]. A study was conducted on rats with hypercholesterolemia to examine their antioxidant levels. The study found no significant change in the levels of ascorbic acid and α-tocopherol after being fed a capsaicin diet [42].

On the first day after dental implants are applied, osteoblasts collapse on the implant surface covered by calcified fibrils and form collagen fibrils from osteoid tissue. Following the implantation process, reparative trabecular bone forms in the surrounding tissue of the implant [43]. Osteoblasts, in addition to being important for the osteogenesis process, induce the production of bone matrix proteins such as collagen type 1α1, osteocalcin, and alkaline phosphatase [44]. The dental implants themselves, their surface, tissue, and type of bone tissue are factors that affect osseointegration. In the osseointegration process, there are periods such as osteophilic, osteoinductive, osteoconductive, and osteoadaptive. The first and second of these periods require a good host bone bed and a maximum internal healing period [45]. In a rat study aiming to examine the effect of capsaicin on bone, bone loss was found to be higher in the capsaicin-free rat group compared to the capsaicin-treated group. Therefore, the current study was designed around the osseointegration process of capsaicin, which alters its healing potential [21]. In a rat study, the most common type of connective tissue formed during the healing phase in animals is fibroblasts, and these immature fibroblasts are extremely important for cell differentiation [46].

In the literature, a study in which capsaicin was given to rats at 125 mg/kg claimed to cause bone loss and reduce bone formation. He explained this situation as being due to the difference in the age or species of the rats.

The literature suggests that the compensatory mechanism during the modeling phase of skeletal growth may interfere with bone formation inhibited by capsaicin treatment. Additionally, it has been reported that capsaicin neuronal ablation has a pro-resorptive effect that can lead to bone loss.

Inflammation is an extremely important mechanism for immunity, and a controlled inflammatory immune response is critical for bone formation, osseointegration, and successful regenerative capacity [47].

TRPV1 is directly related to oxidative stress in many studies, including hippocampus, pain, ischemia-reperfusion, and brain damage [48,49]. We can explain the suppression of oxidative damage in capsaicin groups with TRPV1, which emerged in the study of TRPV1 in capsaicin. The capsaicin active ingredient in vocacapsaicin has been reported to affect TRPV-1 receptors in connection with C fibers after secretion [11,12,50]. In one study, rats were evaluated radiographically after administration of a 0.3 mg/kg medium dose of voxacapsaicin. An increase in bone healing was observed compared to the control group. In studies, TRPV-1 has been reported to play an active role in bone healing in rats and rabbits. In one study, a unilateral femur fracture model was created, and delayed bone healing was observed in the TRPV-1-deficient group [51]. The effect of vocacapsaicin use on bone healing has not been fully elucidated. However, it is currently believed that the TRPV-1 receptor family is involved in the bone healing mechanism [52].

Studies have reported that capsaicin has antioxidant properties. It has also been emphasized that capsaicin has a positive effect on the bone healing mechanism. Similarly, in our study, a significant increase in the number of osteocytes was observed in capsaicin-treated groups. As emphasized in previous studies, we think that this is due to the positive effect of capsaicin on osteogenic activity occurring between TRPV1 and CGRP. In our study, different doses of capsaicin were administered. Bone cells and inflammation, which play a role in bone healing, were evaluated. As a result, an increase in bone cells was observed in the capsaicin groups. There was also a decrease in inflammation. Positive effects were observed in the doses we used. When we researched, we identified the effects of this condition on various factors. Due to the positive effects of these factors, we detected positive effects on bone metabolism at the doses we used. In light of these results, there is a similarity between bone healing and implant osteointegration. Systemic applications of capsaicin may also positively affect this process.

Capsaicin is important in terms of nutrition because it is found in the foods we consume daily and is an herbal product. We think that capsaicin can be used clinically with dose adjustment to improve the osseointegration of dental implant applications, which have become popular in recent years.

Our study has several limitations, especially the limited number of subjects used to avoid killing a large number of animals. Additional studies are needed to obtain more accurate information.

## 5. Conclusions

We conducted this study because we frequently use capsaicin-containing foods, such as hot peppers, in our daily lives. In our study, we concluded that the dose used in previous studies is important, and therefore, we used two different doses. In this study, systemic capsaicin was administered at different doses. Capsaicin has antioxidant activity, but it has not been successful in bone osteointegration. However, capsaicin was observed to affect osteoclasts. We think that this affects the oxidative stress mechanism. More studies are needed for more accurate and reliable results.

## Figures and Tables

**Table 1:** Comparison of changes in serum biochemical values between groups on day 28.

Parameters	Group 1 (*n*)	Group 2 (*n*)	Group 3 (*n*)	*p* † Value	P1–2	P1–3	P2–3
TAS	1.19 ± 0.15 (7)	1.38 ± 0.09 (7)	1.45 ± 0.08 (7)	0.001 **	0.011 *	0.001 **	0.458
TOS	15.21 ± 1.35 (7)	11.81 ± 0.65 (7)	11.11 ± 0.88 (7)	<0.001 **	<0.001 **	<0.001 **	0.410
OSI	1.30 ± 0.20 (7)	0.87 ± 0.05 (7)	0.77 ± 0.07 (7)	<0.001 **	<0.001 **	<0.001 **	0.302

*p* †: Significant difference between groups according to the ANOVA test; *: Statistically significant; **: Strong statistically significant.

**Table 2:** Comparison of changes in capsaicin percentage values.

Groups	Control (M ± SS)	Capsaicin 25 (M ± SS)	Capsaicin 50 (M ± SS)	*p* * Value
Capsaicin	31 ± 2.16	33 ± 2.94	34.29 ± 3.50	0.134

Values are given as the mean (M) ± SD (standard deviation). *p* * value: significance.

**Table 3:** Differences between groups in terms of measurement values.

		Group			Kruskal–Wallis H Test			
		*n*	Mean	Median	Sd	Mean Rank	H	*p*
Fibroblast	Control	7	2.29	2	0.76	9.57	1.667	0.435
	Capsaisin I	7	2.43	2	0.53	10.29		
	Capsaisin II	7	2.71	3	0.49	13.14		
	Total	21	2.48	3	0.6			
Osteoblast proliferation	Control	7	2.43	3	0.79	13.36	1.788	0.409
	Capsaisin I	7	2	2	0.82	9.93		
	Capsaisin II	7	2	2	0.58	9.71		
	Total	21	2.14	2	0.73			
Osteoid matrix formation	Control	7	2	2	0.58	8	3.475	0.176
	Capsaisin I	7	2.43	2	0.53	11.79		
	Capsaisin II	7	2.57	3	0.53	13.21		
	Total	21	2.33	2	0.58			
Inflammatory cell infiltration	Control	7	2.57	3	0.53	14.79	5.469	0.065
	Capsaisin I	7	2	2	1	10.71		
	Capsaisin II	7	1.57	2	0.53	7.5		
	Total	21	2.05	2	0.8			
Hyperemia	Control	7	2.57	3	0.79	14.86	5.591	0.061
	Capsaisin I	7	2	2	0.82	10.64		
	Capsaisin II	7	1.57	2	0.53	7.5		
	Total	21	2.05	2	0.8			

## Data Availability

Data are available upon request to the corresponding author with reasonable cause.
